# SCPortalen: human and mouse single-cell centric database

**DOI:** 10.1093/nar/gkx949

**Published:** 2017-10-17

**Authors:** Imad Abugessaisa, Shuhei Noguchi, Michael Böttcher, Akira Hasegawa, Tsukasa Kouno, Sachi Kato, Yuhki Tada, Hiroki Ura, Kuniya Abe, Jay W Shin, Charles Plessy, Piero Carninci, Takeya Kasukawa

**Affiliations:** Division of Genomic Technologies (DGT), RIKEN Center for Life Science Technologies (CLST), Yokohama, Kanagawa 230-0045, Japan; RIKEN BioResource Center, Tsukuba, Ibaraki 305-0074, Japan

## Abstract

Published single-cell datasets are rich resources for investigators who want to address questions not originally asked by the creators of the datasets. The single-cell datasets might be obtained by different protocols and diverse analysis strategies. The main challenge in utilizing such single-cell data is how we can make the various large-scale datasets to be comparable and reusable in a different context. To challenge this issue, we developed the single-cell centric database ‘SCPortalen’ (http://single-cell.clst.riken.jp/). The current version of the database covers human and mouse single-cell transcriptomics datasets that are publicly available from the INSDC sites. The original metadata was manually curated and single-cell samples were annotated with standard ontology terms. Following that, common quality assessment procedures were conducted to check the quality of the raw sequence. Furthermore, primary data processing of the raw data followed by advanced analyses and interpretation have been performed from scratch using our pipeline. In addition to the transcriptomics data, SCPortalen provides access to single-cell image files whenever available. The target users of SCPortalen are all researchers interested in specific cell types or population heterogeneity. Through the web interface of SCPortalen users are easily able to search, explore and download the single-cell datasets of their interests.

## INTRODUCTION

Single-cell omics recently emerged as a powerful toolset to investigate heterogeneity of large populations of cells with regards to their functions and morphologies (1). Single-cell technologies provide detailed information per biological sample including gene expression profiles and high resolution cell images. Among others, improvements in sequencing, microscopy and microfluidic technologies led to a rapid increase in complex datasets with single-cell resolution. However, lack of a database platform to achieve easy comparison and integration of single-cell data was a great barrier to efficiently investigate and re-use the published results.

Thus, we developed SCPortalen, a single-cell centric database platform. The aim of this database is to provide a gateway to utilize the untapped potential of single-cell dataset. To build the database, we first collected published single-cell transcriptomics data in human and mouse. The datasets which contain raw sequence and metadata have been retrieved from any of the international nucleotide sequence database collaboration (INSDC) data sites (2). The metadata (detailed information) about the biological samples, used protocols and library construction methods are manually curated based on the main publication of each dataset.

Second, to add values to each dataset we developed an analysis pipeline composed of three parts: (i) applying common quality assessment procedures, which enables evaluation and assessment of each dataset in a standardized way; (ii) redoing primary data processing including alignment of raw sequence reads to a reference genome, classification of mapped reads into genomic sub-regions and gene-level expression quantification; and (iii) performing advanced analysis including clustering of cells (principle component analysis (PCA) (3) and t-Distributed Stochastic Neighbor Embedding (t-SNE) (4)), quantification of possible genomic contaminations, functional annotation of expressed genes, cell-cell gene expression correlation and cell-cycle phasing of individual cells.

In addition to the comprehensive metadata and transcriptomics data, SCPortalen also stores cell images and z-stack movies for visual inspection of cell status and quality of captured cells (e.g. identify debris/doublets), whenever such image files have been available.

To integrate all the above mentioned data, we designed a database model to be able to expand the database scheme to accommodate different data types generated by single-cell experiments, and developed a web-based user-interface.

## MATERIALS AND METHODS

### Methods overview

**Figure 1.**
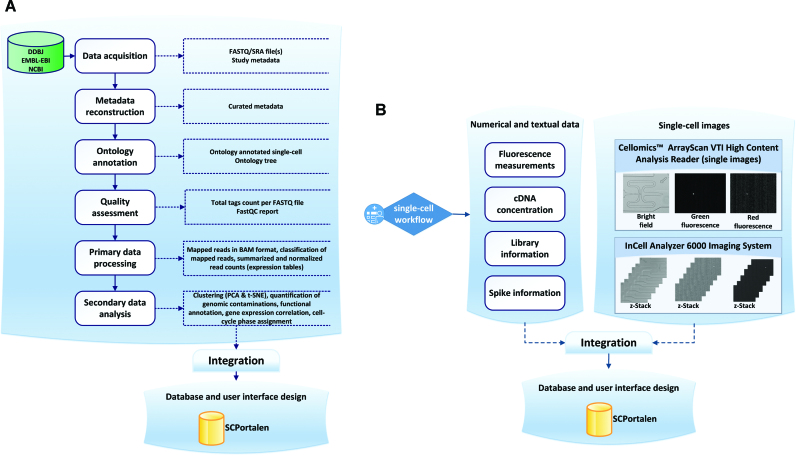
Workflow for data processing. (**A**) General workflow for acquiring, processing and publishing single-cell datasets. The workflow consists of six processes. The main input to the workflow is study accession number. The data acquisition of raw sequence files (FASTQ/SRA) and the study metadata followed by quality assessment procedures, metadata construction and ontology annotation. All outputs are integrated into the SCPortalen database. (**B**) Workflow for integrating single-cell images. Two main microscopic platforms have been used to capture single-cell images.

Our methodology of the data production for SCPortalen is illustrated in (Figure 1). The method in (Figure 1A) used for acquiring, processing and integrating the single-cell transcriptomics data. Figure 1B illustrates the processing of single-cell images and their integration into the database. We applied best practices in user-interface design and information architecture when developing SCPortalen (5).

### Raw sequence and metadata acquisition

We collected published single-cell datasets by searching PubMed for human and mouse single-cell RNA-sequencing articles. Since the single-cell publications and dataset are growing rapidly, our strategy is to include different type of cells generated by different technology platforms. This is to assure that we cover a wide range of cell types and dataset generated by different platforms. From the selected articles, we retrieved study accession number(s) of their original data deposited to INSDC. The study accession numbers were used to retrieve sequence read files and metadata files from any of the INSDC sites (Figure 1A). To obtain FASTQ files, we implemented an automated program using the NCBI SRA Toolkit (6). Each single-cell raw sequence consists of one or two FASTQ file(s) depending on its library construction method. To fetch metadata associated with sequence data for each study accession number, we utilized the Entrez programming utilities (E-utilities) from NCBI (7). The study metadata contains basic information about the biological samples and experimental protocols that authors provided.

### Metadata reconstruction and cell ontology annotation

The downloaded study metadata was reformatted and curated based on the information provided in the methods section and supplementary materials of each main dataset publication. This step generates comprehensive metadata for each study. In addition, we manually assigned standard ontology term(s) to each single-cell sample. The ontology term was selected from the Cell Ontology (8), the Cell Line Ontology (9) or the Uber Anatomy Ontology (Uberon) (10). The ontology term assigned for each cell according to the following rules: if the single-cell originated from cell line, then we assign ontology term from cell line ontology (9), if the cell originated from primary cell then we assign term from cell ontology (8) and if the cell is originated from tissue we assigned term from Uber anatomy ontology (Uberon) (10). In principle, we selected the nearest term from the target ontology based on *is-a* or *part-of* relationship in the tree ontology (11). In case we couldn’t find the matching ontology term from any of the three mentioned ontologies, we look for other ontologies like FMO etc.

### Basic QC, primary data processing

To obtain quality assessment metrics of the raw sequence reads, we performed FastQC tool [http://www.bioinformatics.babraham.ac.uk/projects/fastqc/]. As primary data processing, all FASTQ files were realigned to a recent reference genome build (GRCh38 or GRCm38 genome assembly). We used the STAR software (version 2.5.1b) (12) with default settings and GENCODE gene annotations in the release v24 for human and the release vM9 for mouse. We quantified gene expression counts using featureCounts (in the Subread package Version 1.5.0-p1) (13). The gene expression counts are normalized into transcripts per million reads (TPM) and fragments per kilobase million (FPKM) to generate a gene expression table for each study.

The BAM files and the log files generated by STAR were used to obtain further quality assessment metrics including the total read count, number of uniquely mapped reads and assigned reads (mapped reads assigned to gene) for each single-cell sample. BAM files were also used for classification of the mapped reads into sub regions in the annotated genes (3′ UTR, 5′ UTR, coding exon, intron, intergenic and so on). To estimate possible genomic contamination (e.g. due to the PCR amplifications of the starting material of genomic DNA), we modeled and applied the following formula:

Possibility of genomic contamination (%) = (((total number of mapped reads) − (the total number of reads that are assigned to a gene feature in GENCODE annotation))/(total number of mapped reads)) * 100.

### Types, processing and integration of single-cell image

The Cellomics ArrayScan VTI high content analysis reader was set up to create three images per cell (bright field, green fluorescence and red fluorescence mode). For each Fluidigm C1 run a maximum of 288 images could be generated. The second imaging platform used was InCell Analyzer 6000, which can produce vertical z-stack images (i.e. 11 images) for bright field, green fluorescence and red fluorescence mode. In total InCell Analyzer 6000 produced 3168 TIFF formatted images per Fluidigm C1 medium sized array. We further developed an automated pipeline to compress the TIFF images from 8.1 MB to 188 KB in JPEG format (Supplementary Figure S1). The JPEG format is used to create a z-Stack movie of the 11 z-stack images using ImageMagick version 6.5.4-7.

### Secondary data analysis

We conducted several types of secondary analyses based on the normalized gene expression estimates. In short, the following analysis was performed: (i) PCA and t-SNE analysis, (ii) cell-cycle phase assignment to individual cells and (iii) functional annotation of the highly expressed genes using the DAVID tool (14).

For PCA analysis, we used the prcomp function in R, and for t-SNE analysis we used the Rtsne package in R. The cell-cycle phase assignment is based on the expression profile of the cell and we used the tool and the predefined human cell-cycle gene/marker set provided in (15,16). We obtained the orthologous mouse genes of the above human cell-cycle gene marker set to be able to predict a cell cycle phase for each mouse cell sample.

### Dataset gene expression correlation

**Figure 2.**
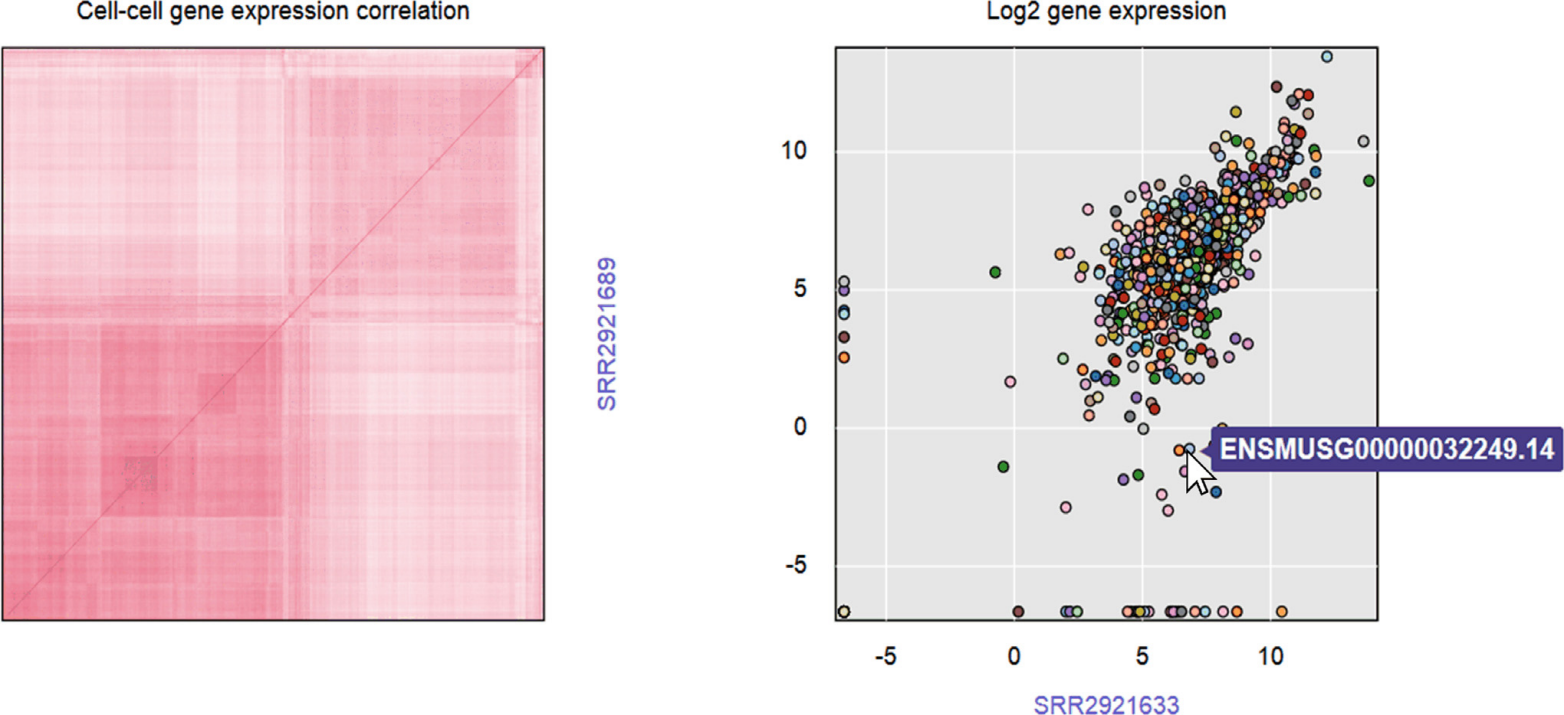
Example of computed gene expression correlation matrix. The correlation matrix as implemented in SCPortalen. It shows the gene expression correlation for the dataset titled Identification of novel regulators of Th17 cell pathogenicity by single-cell genomics (18). In the figure the *y*- and *x*-axis is the cell_id. The color of each dot in the right panel represents the level of the gene expression. When users hover over any dot, its Gencode ID will display.

We created a gene expression correlation matrix for each study. To calculate correlations among samples, the expression tables were pre-processed to replace low TPM values (<0.01) with 0.01 and transformed to log_2_ gene expressions of each TPM value. The qtlcharts R package has been used to create interactive charts to visualize cell–cell gene expression correlations (17). The qtlcharts can generate two views: a heatmap of cell-cell gene expression correlations on the left (columns and rows are cells) and scatterplots of the underlining log_2_ gene expression on the right of the graph. The computed gene expression correlation matrices are stored in SCPortalen (see Figure 2) as an example with a dataset from (18).

### System architecture of SCPortalen

SCPortalen is implemented using PHP (version 5.6.8). We used MySQL server (version 5.6.24) as a back-end database management system. The web server of SCPortalen is Apache version 2.2.15.

## RESULTS

### Single-cell datasets in the SCPortalen

Table 1 shows statistics of the SCPortalen content. At the time of this publication, the database contains 67 146 transcriptomics profiles of human and mouse cells with curated metadata. In addition, the database contains about 36 000 single-cell images and z-stack movies. Overall, the database covers 78 human cell types (Supplementary Figure S2) and 119 mouse cell types (Supplementary Figure S3). The datasets in SCPortalen are divided into two sections, the single-cell transcriptomics datasets and the single-cell images.

**Table 1.** Count statistics of SCPortalen database content

Attribute	Organism	
	Homo sapiens	Mus musculus
Number of single-cells	20 761	46 385
Number of datasets	23	47
Number of cell types	79	119
Number of ontology terms	67	85
Number of FASTQ files	61 938	
Number of BAM files	60 217	
Number of cell images	32 256	0
Number of z-stack movies	5412	0

This table shows general statistics of the content and coverage of the SCPortalen.

### User-interface to the SCPortalen

We designed the SCPortalen interface to enable users to navigate through and be able to perform basic operations, such as, searching, querying and downloading data. The user-interface is divided into sub-views as explained below. The online user guide in SCPortalen illustrates several use-cases of available views.

#### Single-cell studies view

**Figure 3.**
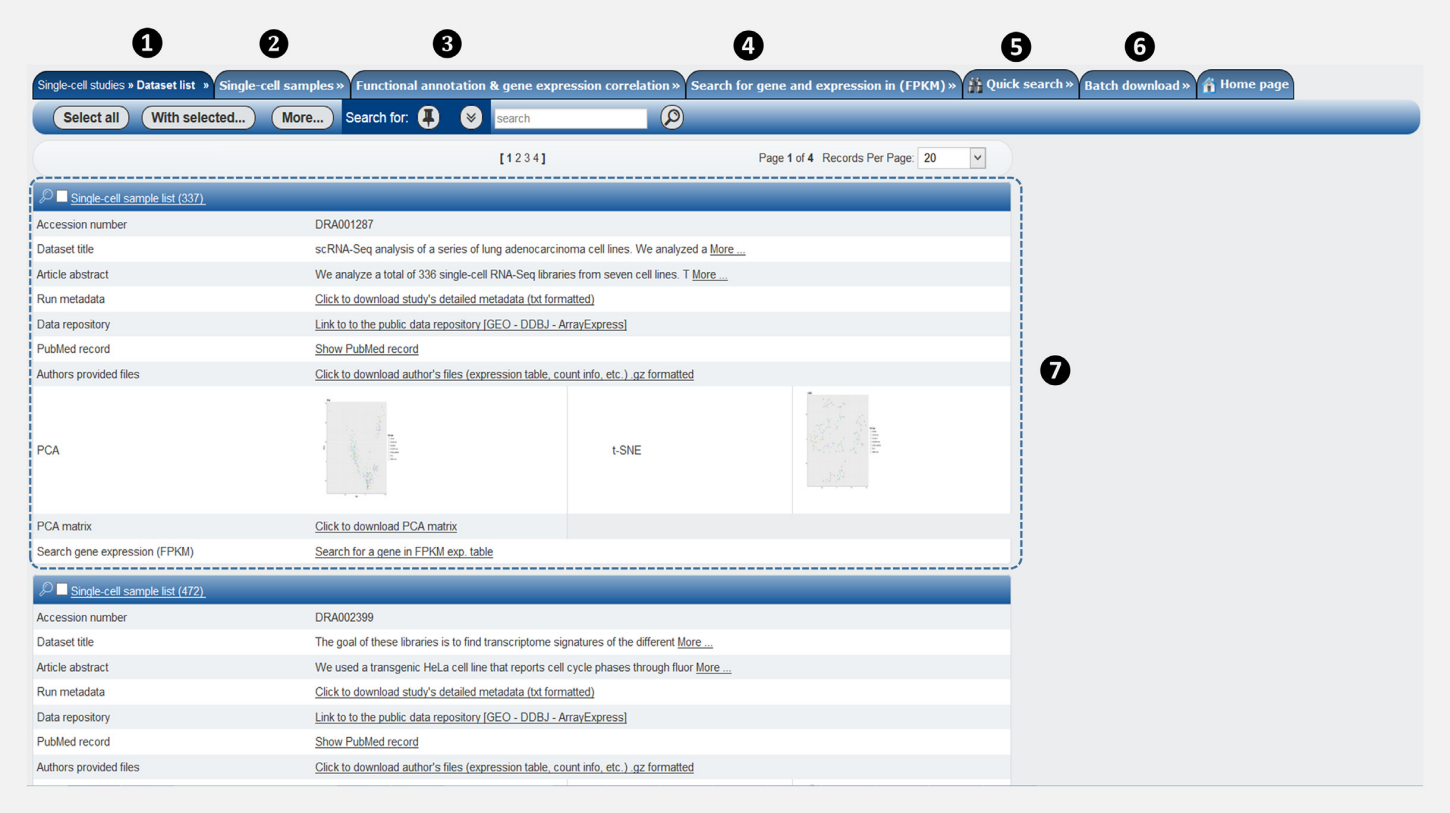
Single-cell transcriptomics dataset view. The figure shows the main elements of the single-cell transcriptomics dataset view. In the main menu bar, (1)–(6). The single-cell studies view is the active tab (7). For each study, a summary of the total number of samples is shown at the top left corner of the rectangle. Several attributes and links are provided in addition to the PCA and t-SNE plots. We also provide the computed PCA matrix (PC computed in FPKM) for each dataset as a table for downloading from this view.

This view gives a general description of each single-cell study in SCPortalen (see Figure 3). It provides the following information: (i) the study accession number of the dataset in INSDC (e.g. GSE48968), (ii) the dataset title which summarizes the type of the dataset and how it has been generated, (iii) the abstract of the associated main article publication, (iv) the curated metadata for downloading as a text file, (v) the link to the public data repository, (vi) the link to the PubMed record of the published article and (vii) the author provided metadata files (raw files before curation) for downloading. Moreover, the view shows PCA and t-SNE plots of all single-cell samples in the dataset as well as a computed PCA matrix table with columns 1: sample ID, 2: PC1, 3: PC2, 4: PC3 and 5: cell type. The single-cell studies view enables users to search for a dataset using an accession number or words/terms in the abstract/dataset title. Users are able to access detailed information of each cell sample from this view directly.

#### Single-cell samples view

**Figure 4.**
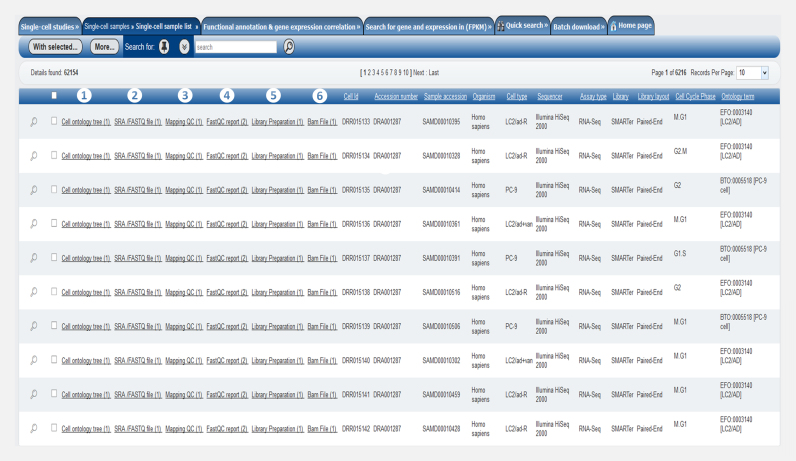
Single-cell samples view. This figure shows the elements of the single-cell sample view. Each sample has a set of attributes and links. (1) The links lead to a cell ontology tree and (2) SRA/FASTQ files on external sites. The ‘Cell ontology tree’ directs to the EBI ontology lookup services (OLS) via web service. The ‘SRA/FASTQ file’ link opens a page for downloading sequence reads files. Other types of links enable direct file downloads, or lead to reports from SCPortalen (e.g. (4) FastQC report, (6) BAM files). Finally, (3) mapping QC and (5) library preparation information are listed in the table view. Using the check-boxes in the left side of the menu user will be able to select any number of cell and export the selected ones to CSV or Excel file format.

This is the most detailed view of an individual cell. Each single-cell sample is assigned 11 attributes described in (Table 2). Each cell sample is also assigned a unique identifier (primary key is cell_id e.g. SRR940235). The cell_id links each single-cell sample to its metadata, a set of files and analysis results (Figure 4), which are available in this view. SCPortalen provides the following links for downloading: (i) the FASTQ/SRA file(s) of the cell in the INSDC repository, (ii) the STAR alignment BAM files and (iii) the FastQC reports. Pre-computed values of the predicted cell-cycle phases and mapping QCs are also shown in this view. Users can search for single-cell sample(s) using one or more keywords against metadata attributes in (Table 2).

**Table 2.** Basic metadata attributes of each single-cell in SCPortalen

Attribute	Description
Cell ID	A unique cell identification number based on the run accession number or the Fluidigm C1 chip ID plus the position information of a cell on the cDNA harvest plate
Accession number	This is a unique study identifier, this accession number provided by INSDEC
Sample accession	This is a unique sample identifier, the sample accession number provided by INSDEC
Organism	This attribute holds the name of the organism in which the single-cell originated from
Cell type	The cell type information as provided by the study authors
Sequencer	The sequencer attribute refers to the platform used to perform the scRNA-seq
Assay type	This field holds the type of assay used for single-cell sample preparation
Library	The library field defines the library protocol used to generate the single-cell library for RNA sequencing
Library layout	This attribute provides layout information of the sequence library, either Single-End or Paired-End
Cell-cycle phase	This is predicted cell-cycle phase based on the transcriptomic profile of the cell. The phases are [G2, G2.M, M.G1, G1.S, S]
Ontology term	The ontology term used to annotate single-cells e.g. CL:0002322 [embryonic stem cell]

This table lists the basic metadata attributes of the single-cell as implemented in SCPortalen database (under the single-cell sample list).

#### Exploring and searching expressed genes

In SCPortalen, expression tables are searchable. The gene search view provides an intuitive interface to search for genes of interest and retrieve all single-cell samples which expressed the gene. The search argument can either be the GENCODE Gene ID (e.g. ENSG00000000003.14) or the GENCODE Gene Symbol (e.g. TSPAN6).

#### Data download

SCPortalen enables to download datasets via different methods. Users can select one cell or a group of cells from the single-cell samples view, and export the result. For the cell images data, SCPortalen provides access to all images in a compressed file format for download. The batch download menu provides the user a list of URL addresses to utilize the wget command (wget -A txt,gz -m -p -E -k -K –np **<***URL address****>***) to download whole sets of BAM files, FastQC reports and expression tables for one or multiple studies.

### Curation of metadata

#### Library information

Information about the sequencing library construction protocol is important for investigating batch effects and the quality of sequence data. To provide the library information in a unified format, we manually curated single-cell library preparation metadata. Supplementary Figure S4 summarizes single-cell isolation methods. Supplementary Figures S5 and 6 give an overview of the library preparation protocols and kits. Supplementary Figures S7 and 8 show the total number of single-cell samples in each study for human and mouse subsequently.

#### Ontology annotation

To be able to precisely identify cell types in the database, we performed manual ontology annotation to assign an ontology term to each individual cell (see ‘Materials and Methods’ section). The ontology term can be used to navigate the ontology tree and to see the relationship between the term and other terms in the same ontology hierarchy (11). The summary of the ontology terms annotated to the human and mouse single-cell samples in our database is shown in (Supplementary Figures S9 and 10) subsequently. As an example, the cell ontology term for the cell type ‘LC2/AD’ is ‘EFO_0003140’ that has the following ontological relationships with other ontology terms: (i) ‘*is-a’* Homo sapiens cell line (EFO_0002888), (ii) ‘*is-a’* Lung cancer cell line (EFO_0002934) and (iii) ‘*bearer_of’* lung adenocarcinoma (EFO_0000571). Users are able to navigate and explore the above relationships by using the EBI ontology-lookup services (19).

### Quality assessment metrics in the database

SCPortalen stores and provides access to quality assessment results in the form of FastQC reports and a list of mapping QC metrics. The FastQC report contains detailed information about sequence reads and a summary of their quality with some statistics and graphs. The mapping QC metrics include: (i) classification of the mapped read, (ii) percent of uniquely mapped reads (as percentage of total read count), (iii) percent of reads mapped to multiple loci, (iv) percent of reads mapped to multiple loci and (v) percent of possible genomic contamination.

### Further analysis for single-cell transcriptomics data

#### Cell-cycle phase assignment

Since each single-cell sample can be in a different cell-cycle phase, and this may cause expression differences among single-cell samples. Thus, we assigned a cell-cycle phase for each single-cell sample as an additional assessment for single-cell samples. This information is available in the database.

#### DAVID functional annotation

We compared total FPKM of all samples in each dataset, then we selected broadly expressed 250 genes. The selected genes are used for DAVID functional annotations. Via the dataset views, we provided links to an annotation report and chart using the DAVID bioinformatics tool API (14) [https://david.ncifcrf.gov/]. DAVID provides enrichment analysis to highlight the most relevant annotation category associated with a gene list. The functional annotation and enrichment show how the top expressed genes in each dataset are annotated and provide means to help interpretation of the biological role of the expressed genes.

## CONCLUSION

SCPortalen is the first single-cell database that provides comprehensively curated metadata and analysis results of publicly available single-cell dataset. Furthermore, we attempted to make these datasets comparable by using a unified analysis pipeline.

Additional work will focus on analysis, such as (i) characterization of expression distribution and identification of multi-state genes, and (ii) expression of long non-coding RNA. The database design of SCPortalen will be scale-up to meet the increasing demands of single-cell omics research. Future database update will include processing of whole genome sequence of single-cell and ATAC-Seq, and FISH images. We believe that SCPortalen will be a useful resource for the single-cell research community.

## AVAILABILITY

SCPortalen is accessible from http://single-cell.clst.riken.jp

## Supplementary Material

Supplementary Data
