# Supplementary material for: SCPortalen: human and mouse single-cell centric database
Source: Nucleic Acids Res. 2017 Oct 17;46(Database issue):D781–7. doi: 10.1093/nar/gkx949 (PMC5753281; doi:10.1093/nar/gkx949)
Supplement: Supplementary Data [file gkx949_supp.zip › nar-02451-data-e-2017-File009.pdf]

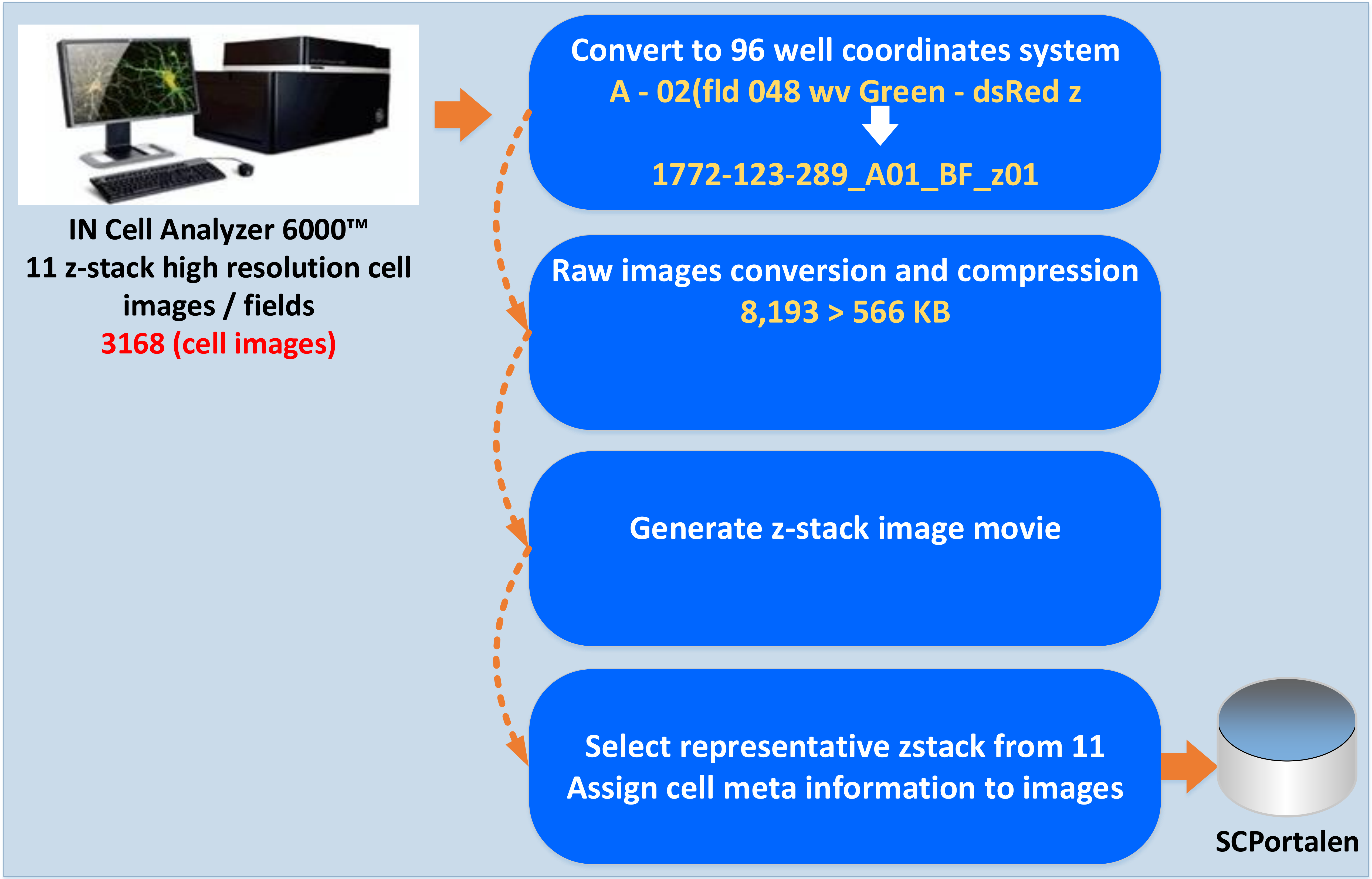

IN Cell Analyzer 6000™
11 z-stack high resolution cell images / fields
3168 (cell images)
Convert to 96 well coordinates system
A - 02(fld 048 wv Green - dsRed z
1772-123-289_A01_BF_z01
Raw images conversion and compression
8,193 > 566 KB
Generate z-stack image movie
Select representative zstack from 11
Assign cell meta information to images
SCPortalen
