# Supplementary material for: SCPortalen: human and mouse single-cell centric database
Source: Nucleic Acids Res. 2017 Oct 17;46(Database issue):D781–7. doi: 10.1093/nar/gkx949 (PMC5753281; doi:10.1093/nar/gkx949)
Supplement: Supplementary Data [file gkx949_supp.zip › nar-02451-data-e-2017-File013.pdf]

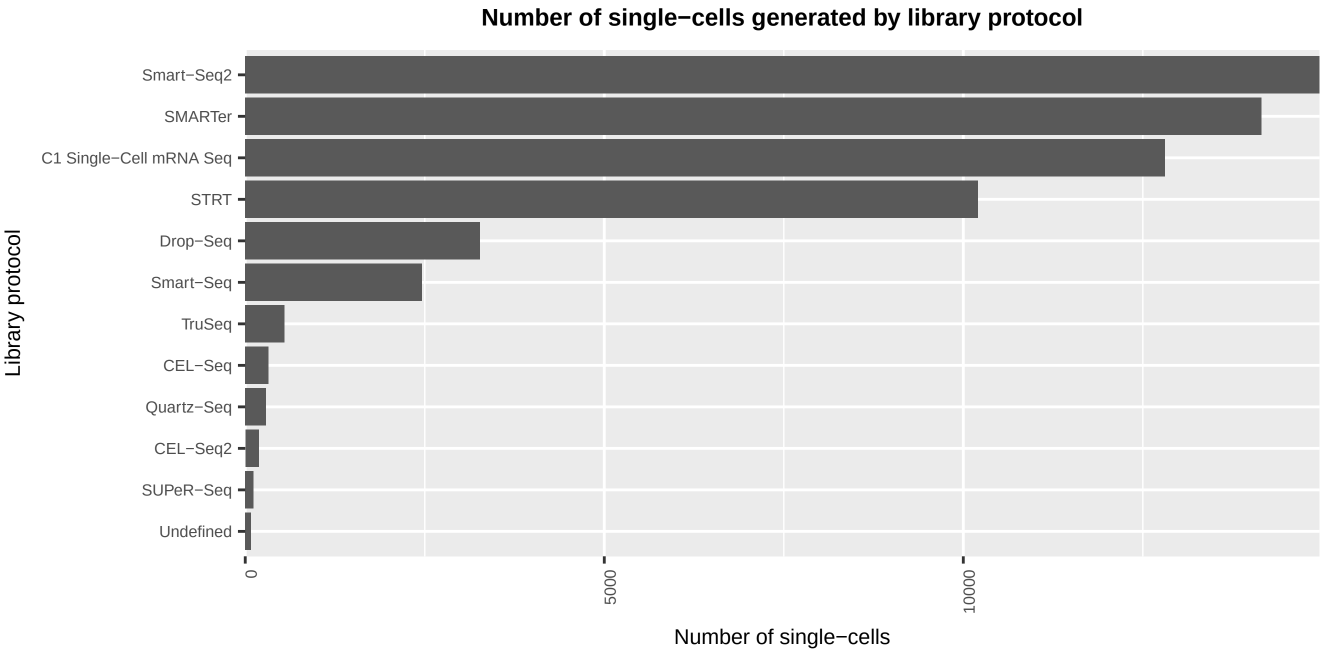
Number of single−cells generated by library protocol
Library protocol
Smart−Seq2
SMARTer
C1 Single−Cell mRNA Seq
STRT
Drop−Seq
Smart−Seq
TruSeq
CEL−Seq
Quartz−Seq
CEL−Seq2
SUPeR−Seq
Undefined
0
5000
10000
Number of single−cells

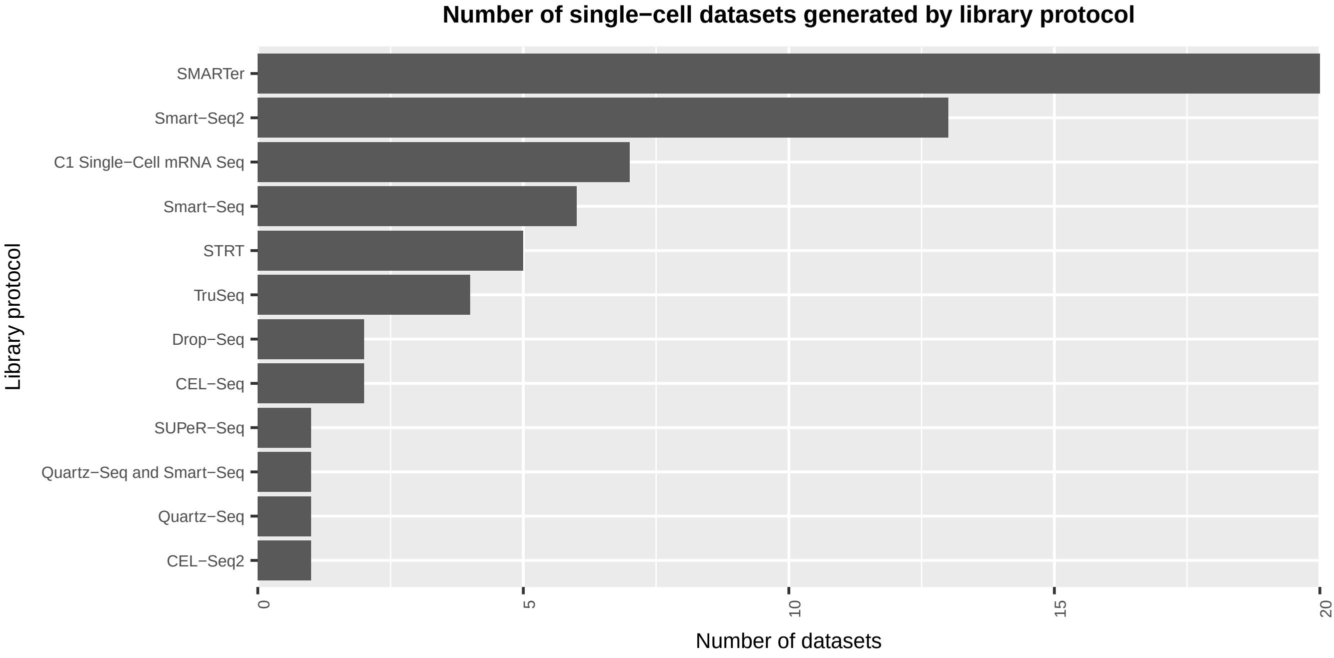
Number of single−cell datasets generated by library protocol
Library protocol
SMARTer
Smart−Seq2
C1 Single−Cell mRNA Seq
Smart−Seq
STRT
TruSeq
Drop−Seq
CEL−Seq
SUPeR−Seq
Quartz−Seq and Smart−Seq
Quartz−Seq
CEL−Seq2
0
5
10
15
20
Number of datasets
