# Supplementary material for: SCPortalen: human and mouse single-cell centric database
Source: Nucleic Acids Res. 2017 Oct 17;46(Database issue):D781–7. doi: 10.1093/nar/gkx949 (PMC5753281; doi:10.1093/nar/gkx949)
Supplement: Supplementary Data [file gkx949_supp.zip › nar-02451-data-e-2017-File016.pdf]

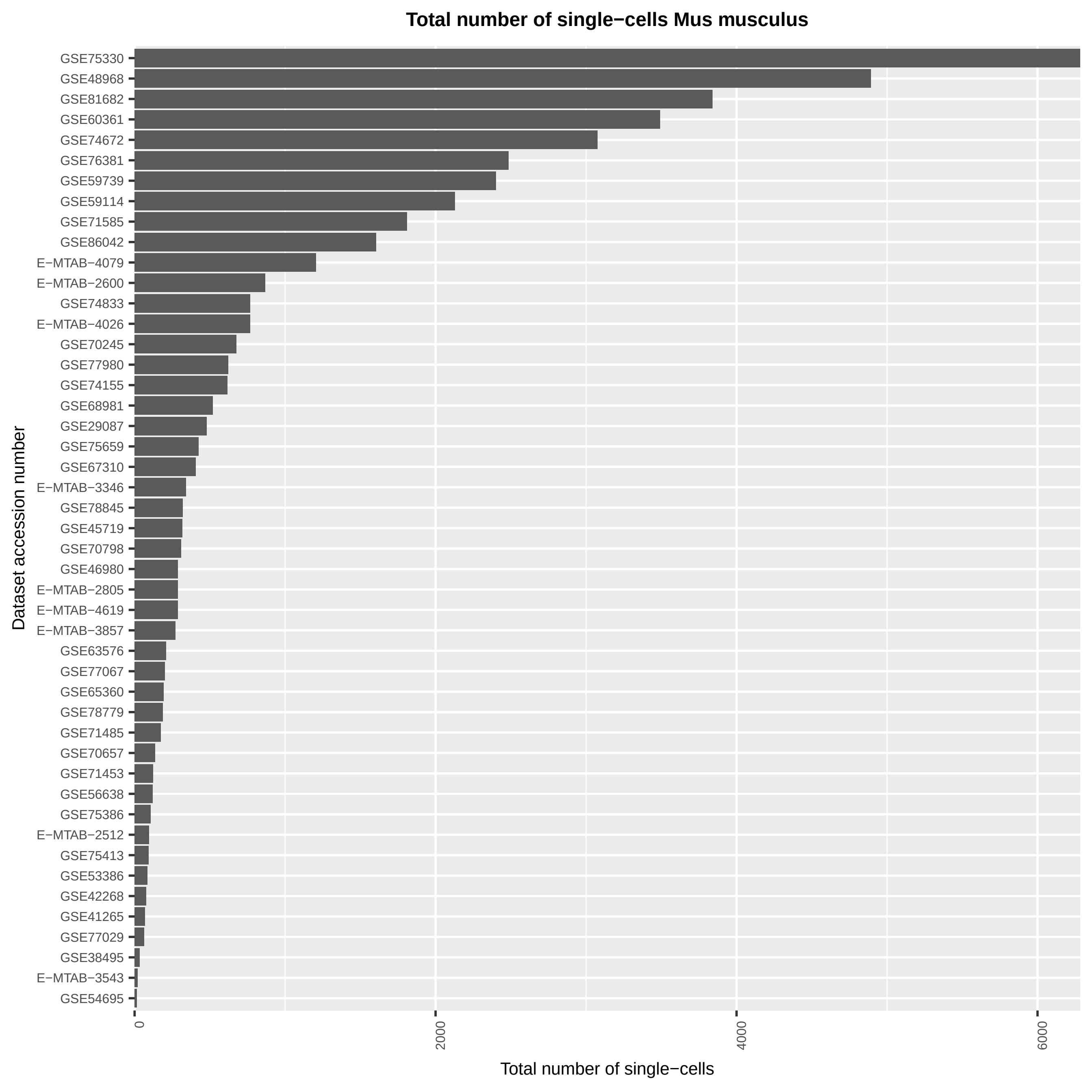

Total number of single−cells Mus musculus
Dataset accession number
GSE75330
GSE48968
GSE81682
GSE60361
GSE74672
GSE76381
GSE59739
GSE59114
GSE71585
GSE86042
E−MTAB−4079
E−MTAB−2600
GSE74833
E−MTAB−4026
GSE70245
GSE77980
GSE74155
GSE68981
GSE29087
GSE75659
GSE67310
E−MTAB−3346
GSE78845
GSE45719
GSE70798
GSE46980
E−MTAB−2805
E−MTAB−4619
E−MTAB−3857
GSE63576
GSE77067
GSE65360
GSE78779
GSE71485
GSE70657
GSE71453
GSE56638
GSE75386
E−MTAB−2512
GSE75413
GSE53386
GSE42268
GSE41265
GSE77029
GSE38495
E−MTAB−3543
GSE54695
0
2000
4000
6000
Total number of single−cells
