# Supplementary material for: SCPortalen: human and mouse single-cell centric database
Source: Nucleic Acids Res. 2017 Oct 17;46(Database issue):D781–7. doi: 10.1093/nar/gkx949 (PMC5753281; doi:10.1093/nar/gkx949)
Supplement: Supplementary Data [file gkx949_supp.zip › nar-02451-data-e-2017-File017.pdf]

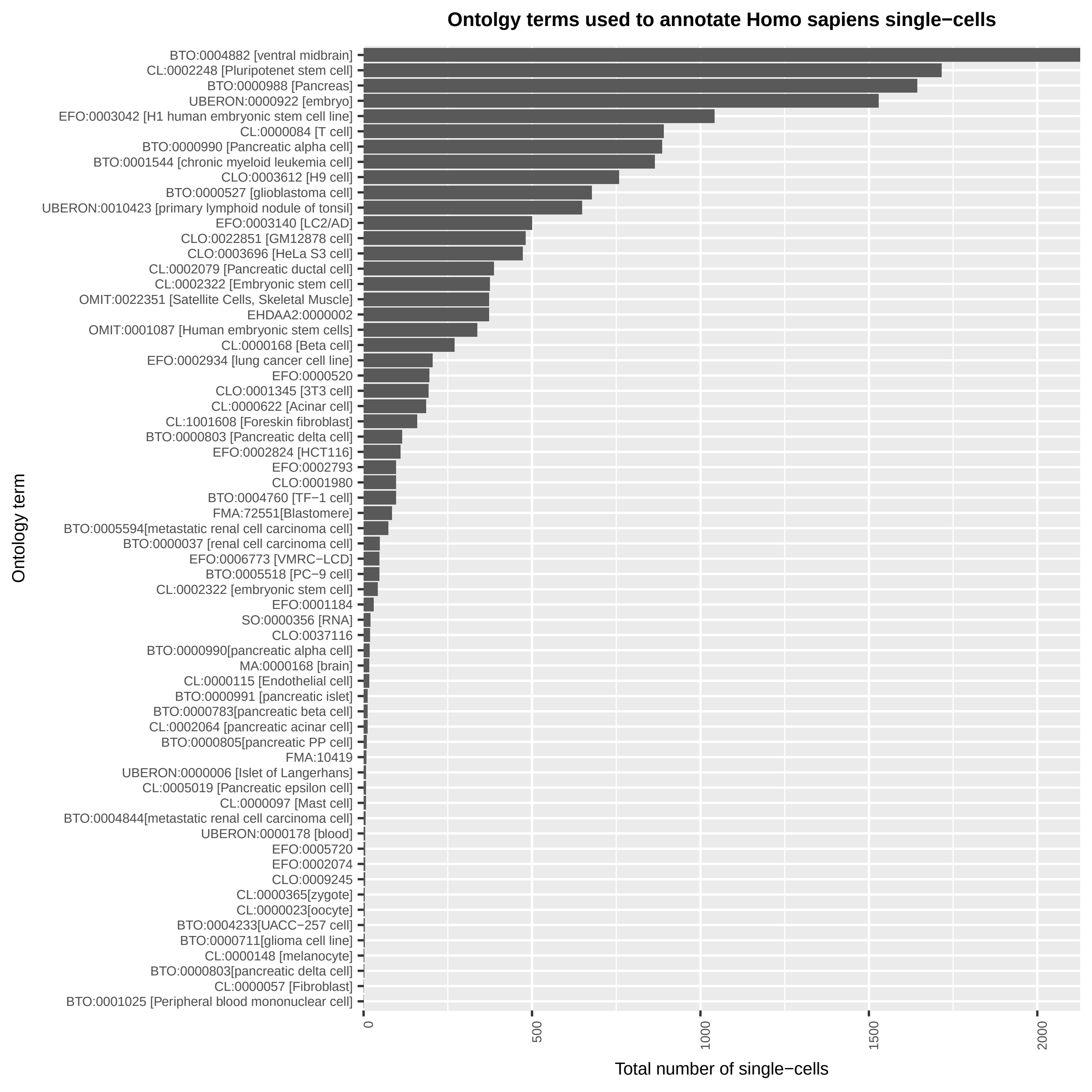

Ontolgy terms used to annotate Homo sapiens single−cells
Ontology term
BTO:0004882 [ventral midbrain]
CL:0002248 [Pluripotenet stem cell]
BTO:0000988 [Pancreas]
UBERON:0000922 [embryo]
EFO:0003042 [H1 human embryonic stem cell line]
CL:0000084 [T cell]
BTO:0000990 [Pancreatic alpha cell]
BTO:0001544 [chronic myeloid leukemia cell]
CLO:0003612 [H9 cell]
BTO:0000527 [glioblastoma cell]
UBERON:0010423 [primary lymphoid nodule of tonsil]
EFO:0003140 [LC2/AD]
CLO:0022851 [GM12878 cell]
CLO:0003696 [HeLa S3 cell]
CL:0002079 [Pancreatic ductal cell]
CL:0002322 [Embryonic stem cell]
OMIT:0022351 [Satellite Cells, Skeletal Muscle]
EHDAA2:0000002
OMIT:0001087 [Human embryonic stem cells]
CL:0000168 [Beta cell]
EFO:0002934 [lung cancer cell line]
EFO:0000520
CLO:0001345 [3T3 cell]
CL:0000622 [Acinar cell]
CL:1001608 [Foreskin fibroblast]
BTO:0000803 [Pancreatic delta cell]
EFO:0002824 [HCT116]
EFO:0002793
CLO:0001980
BTO:0004760 [TF−1 cell]
FMA:72551[Blastomere]
BTO:0005594[metastatic renal cell carcinoma cell]
BTO:0000037 [renal cell carcinoma cell]
EFO:0006773 [VMRC−LCD]
BTO:0005518 [PC−9 cell]
CL:0002322 [embryonic stem cell]
EFO:0001184
SO:0000356 [RNA]
CLO:0037116
BTO:0000990[pancreatic alpha cell]
MA:0000168 [brain]
CL:0000115 [Endothelial cell]
BTO:0000991 [pancreatic islet]
BTO:0000783[pancreatic beta cell]
CL:0002064 [pancreatic acinar cell]
BTO:0000805[pancreatic PP cell]
FMA:10419
UBERON:0000006 [Islet of Langerhans]
CL:0005019 [Pancreatic epsilon cell]
CL:0000097 [Mast cell]
BTO:0004844[metastatic renal cell carcinoma cell]
UBERON:0000178 [blood]
EFO:0005720
EFO:0002074
CLO:0009245
CL:0000365[zygote]
CL:0000023[oocyte]
BTO:0004233[UACC−257 cell]
BTO:0000711[glioma cell line]
CL:0000148 [melanocyte]
BTO:0000803[pancreatic delta cell]
CL:0000057 [Fibroblast]
BTO:0001025 [Peripheral blood mononuclear cell]
0
500
1000
1500
2000
Total number of single−cells
