# Supplementary material for: SCPortalen: human and mouse single-cell centric database
Source: Nucleic Acids Res. 2017 Oct 17;46(Database issue):D781–7. doi: 10.1093/nar/gkx949 (PMC5753281; doi:10.1093/nar/gkx949)
Supplement: Supplementary Data [file gkx949_supp.zip › nar-02451-data-e-2017-File018.pdf]

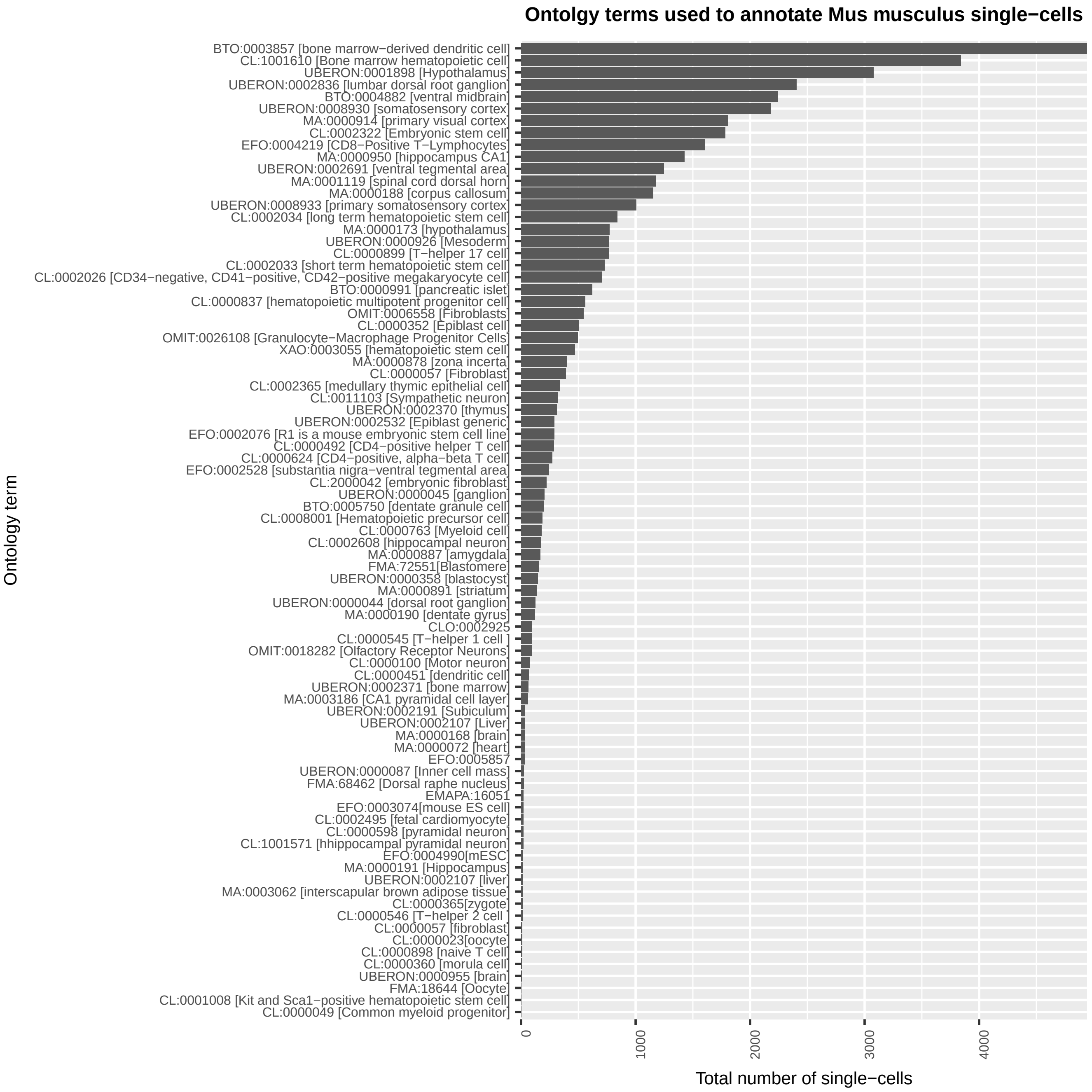

Ontolgy terms used to annotate Mus musculus single−cells
Ontology term
Total number of single−cells
0
1000
2000
3000
4000
BTO:0003857 [bone marrow−derived dendritic cell]
CL:1001610 [Bone marrow hematopoietic cell]
UBERON:0001898 [Hypothalamus]
UBERON:0002836 [lumbar dorsal root ganglion]
BTO:0004882 [ventral midbrain]
UBERON:0008930 [somatosensory cortex]
MA:0000914 [primary visual cortex]
CL:0002322 [Embryonic stem cell]
EFO:0004219 [CD8−Positive T−Lymphocytes]
MA:0000950 [hippocampus CA1]
UBERON:0002691 [ventral tegmental area]
MA:0001119 [spinal cord dorsal horn]
MA:0000188 [corpus callosum]
UBERON:0008933 [primary somatosensory cortex]
CL:0002034 [long term hematopoietic stem cell]
MA:0000173 [hypothalamus]
UBERON:0000926 [Mesoderm]
CL:0000899 [T−helper 17 cell]
CL:0002033 [short term hematopoietic stem cell]
CL:0002026 [CD34−negative, CD41−positive, CD42−positive megakaryocyte cell]
BTO:0000991 [pancreatic islet]
CL:0000837 [hematopoietic multipotent progenitor cell]
OMIT:0006558 [Fibroblasts]
CL:0000352 [Epiblast cell]
OMIT:0026108 [Granulocyte−Macrophage Progenitor Cells]
XAO:0003055 [hematopoietic stem cell]
MA:0000878 [zona incerta]
CL:0000057 [Fibroblast]
CL:0002365 [medullary thymic epithelial cell]
CL:0011103 [Sympathetic neuron]
UBERON:0002370 [thymus]
UBERON:0002532 [Epiblast generic]
EFO:0002076 [R1 is a mouse embryonic stem cell line]
CL:0000492 [CD4−positive helper T cell]
CL:0000624 [CD4−positive, alpha−beta T cell]
EFO:0002528 [substantia nigra−ventral tegmental area]
CL:2000042 [embryonic fibroblast]
UBERON:0000045 [ganglion]
BTO:0005750 [dentate granule cell]
CL:0008001 [Hematopoietic precursor cell]
CL:0000763 [Myeloid cell]
CL:0002608 [hippocampal neuron]
MA:0000887 [amygdala]
FMA:72551[Blastomere]
UBERON:0000358 [blastocyst]
MA:0000891 [striatum]
UBERON:0000044 [dorsal root ganglion]
MA:0000190 [dentate gyrus]
CLO:0002925
CL:0000545 [T−helper 1 cell]
OMIT:0018282 [Olfactory Receptor Neurons]
CL:0000100 [Motor neuron]
CL:0000451 [dendritic cell]
UBERON:0002371 [bone marrow]
MA:0003186 [CA1 pyramidal cell layer]
UBERON:0002191 [Subiculum]
UBERON:0002107 [Liver]
MA:0000168 [brain]
MA:0000072 [heart]
EFO:0005857
UBERON:0000087 [Inner cell mass]
FMA:68462 [Dorsal raphe nucleus]
EMAPA:16051
EFO:0003074[mouse ES cell]
CL:0002495 [fetal cardiomyocyte]
CL:0000598 [pyramidal neuron]
CL:1001571 [hhippocampal pyramidal neuron]
EFO:0004990[mESC]
MA:0000191 [Hippocampus]
UBERON:0002107 [liver]
MA:0003062 [interscapular brown adipose tissue]
CL:0000365[zygote]
CL:0000546 [T−helper 2 cell]
CL:0000057 [fibroblast]
CL:0000023[oocyte]
CL:0000898 [naive T cell]
CL:0000360 [morula cell]
UBERON:0000955 [brain]
FMA:18644 [Oocyte]
CL:0001008 [Kit and Sca1−positive hematopoietic stem cell]
CL:0000049 [Common myeloid progenitor]
