# Supplementary figures and images for: SCPortalen: human and mouse single-cell centric database
Source: Nucleic Acids Res. 2017 Oct 17;46(Database issue):D781–7. doi: 10.1093/nar/gkx949 (PMC5753281; doi:10.1093/nar/gkx949)

# Single-cell types homo sapiens

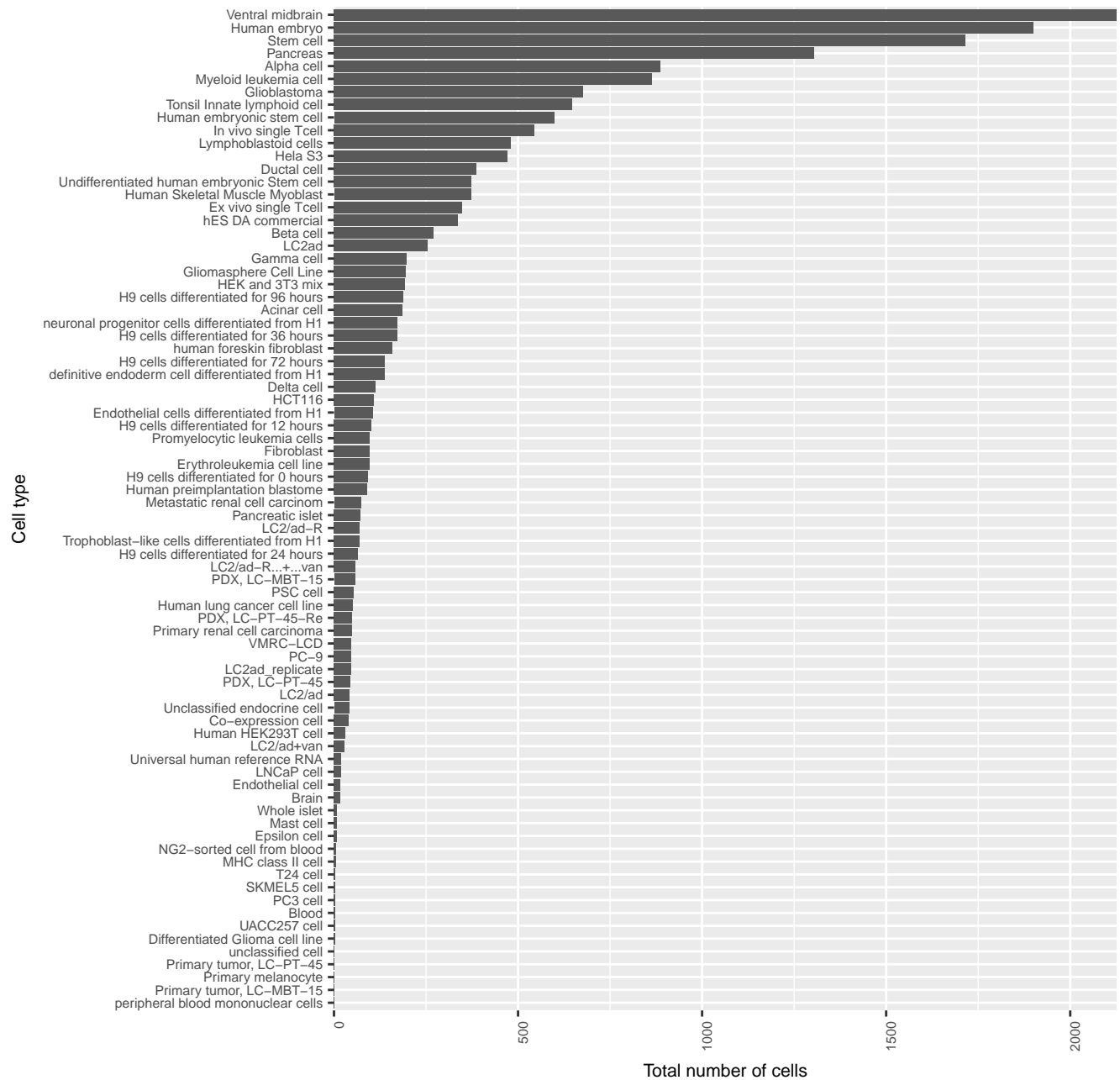

Supplement: Supplementary Data [file gkx949_supp.zip › nar-02451-data-e-2017-File010.pdf]

Single-cell types mus musculus

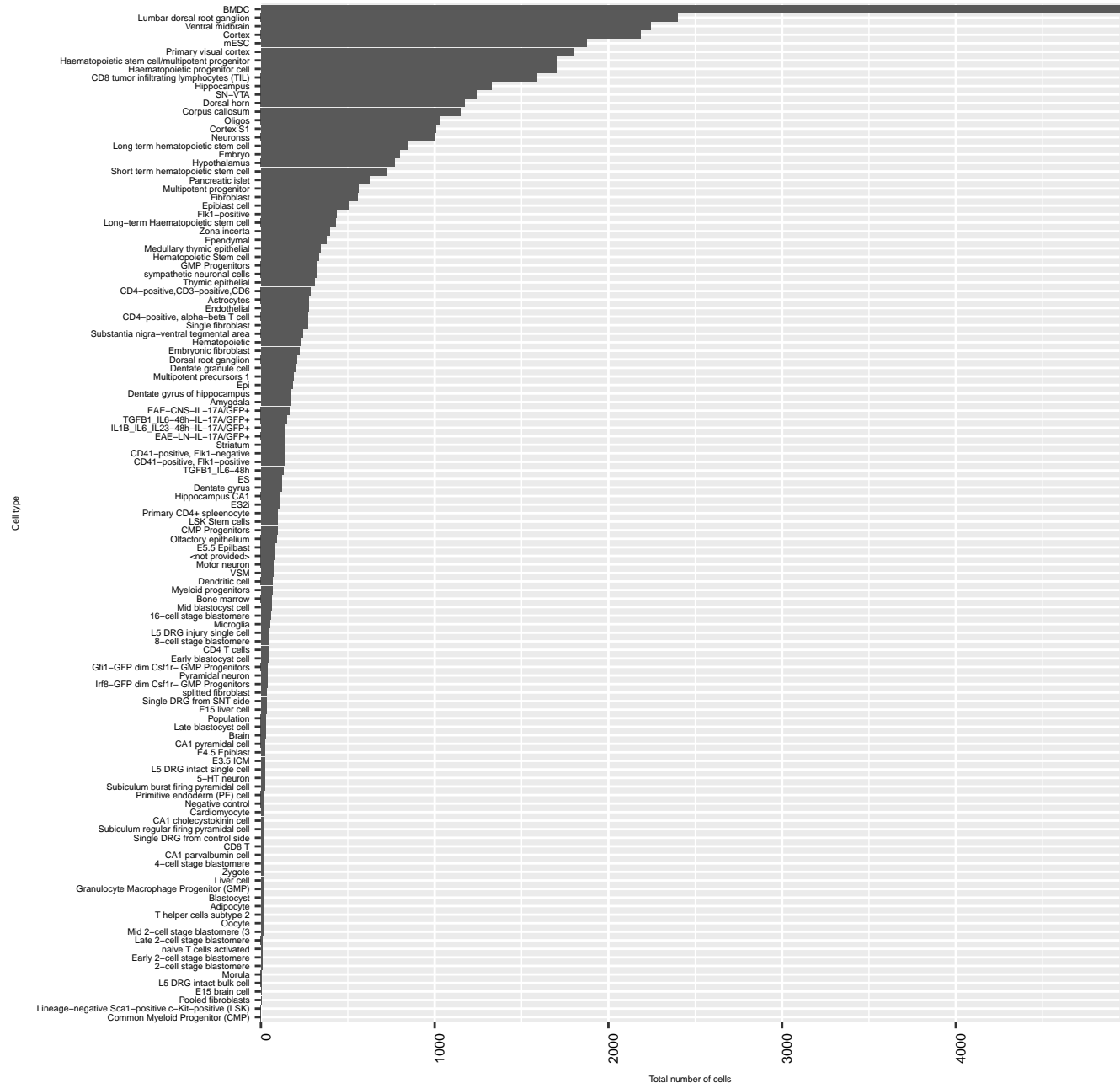

Supplement: Supplementary Data [file gkx949_supp.zip › nar-02451-data-e-2017-File011.pdf]

Number of single-cells isolated by isolation method

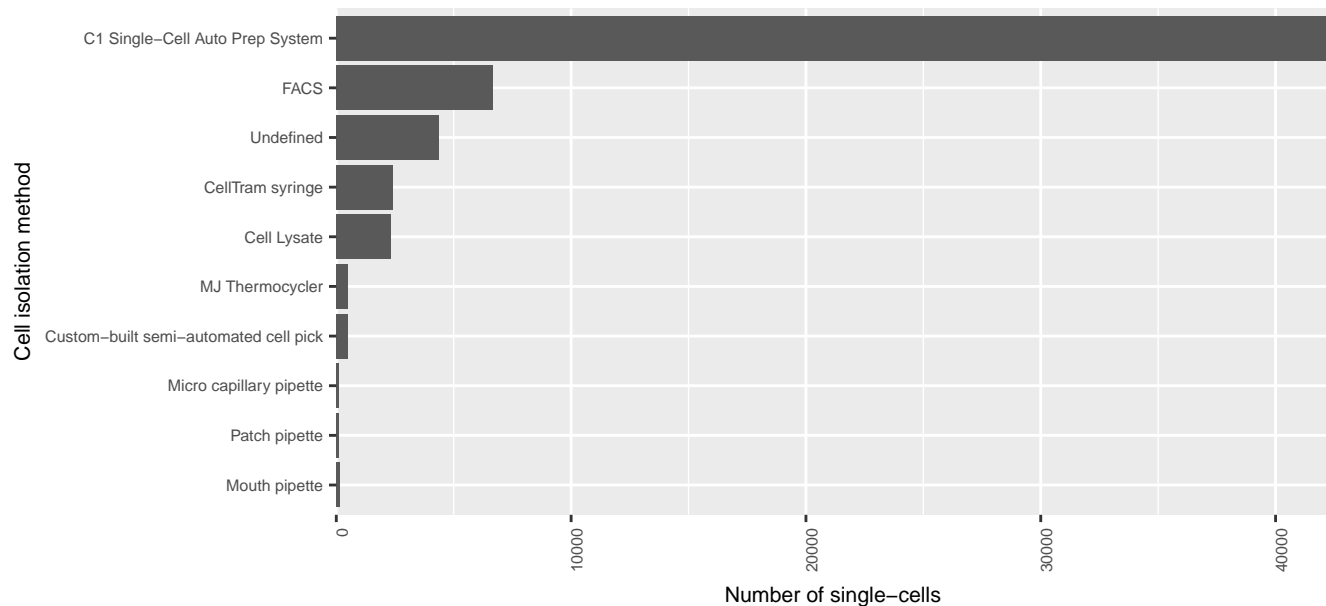

Number of datasets per isolation method

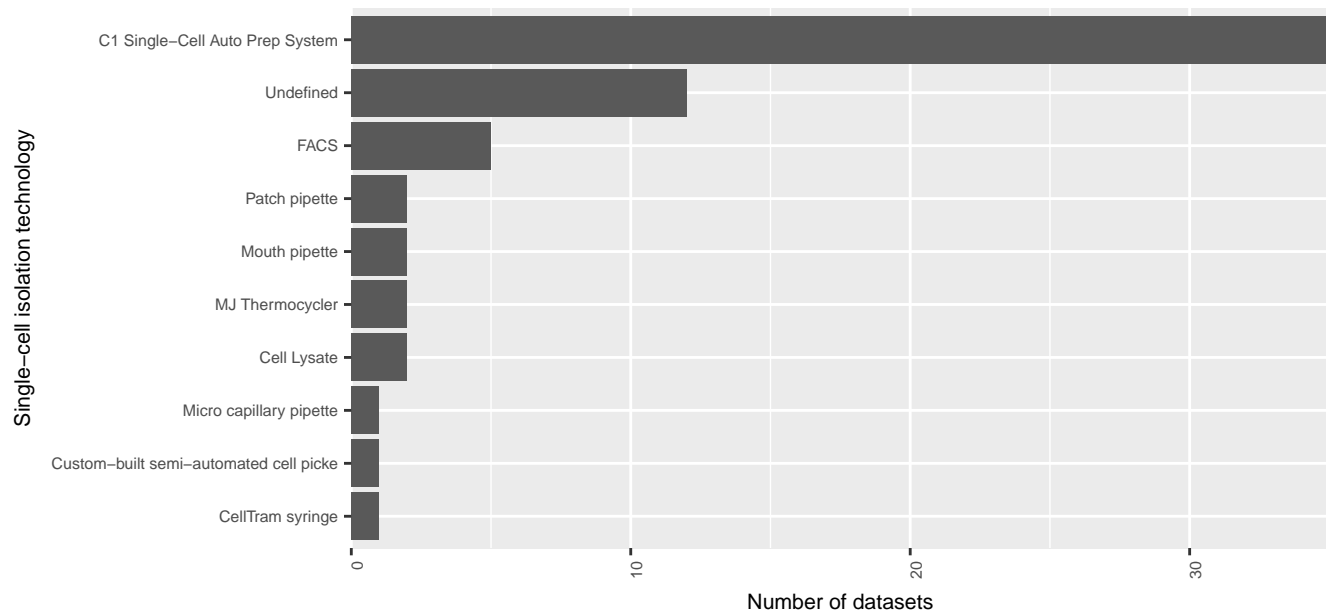

Supplement: Supplementary Data [file gkx949_supp.zip › nar-02451-data-e-2017-File012.pdf]

### Single-cell generated by library preparation kit

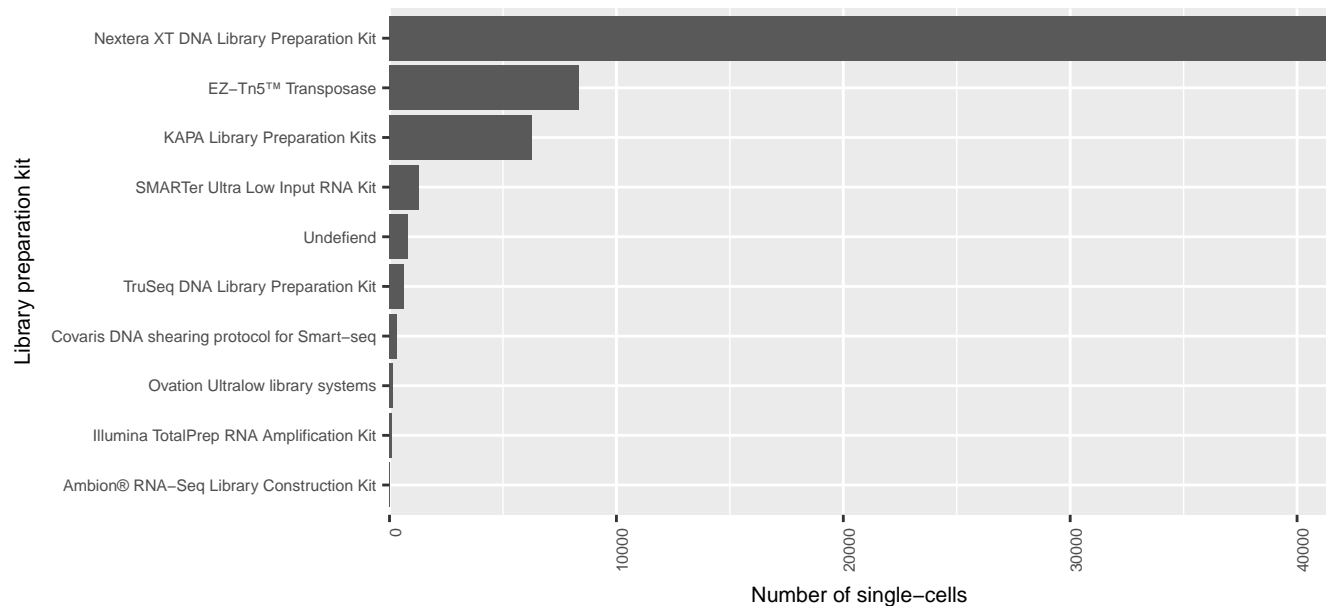

### Library preparation kit used per dataset

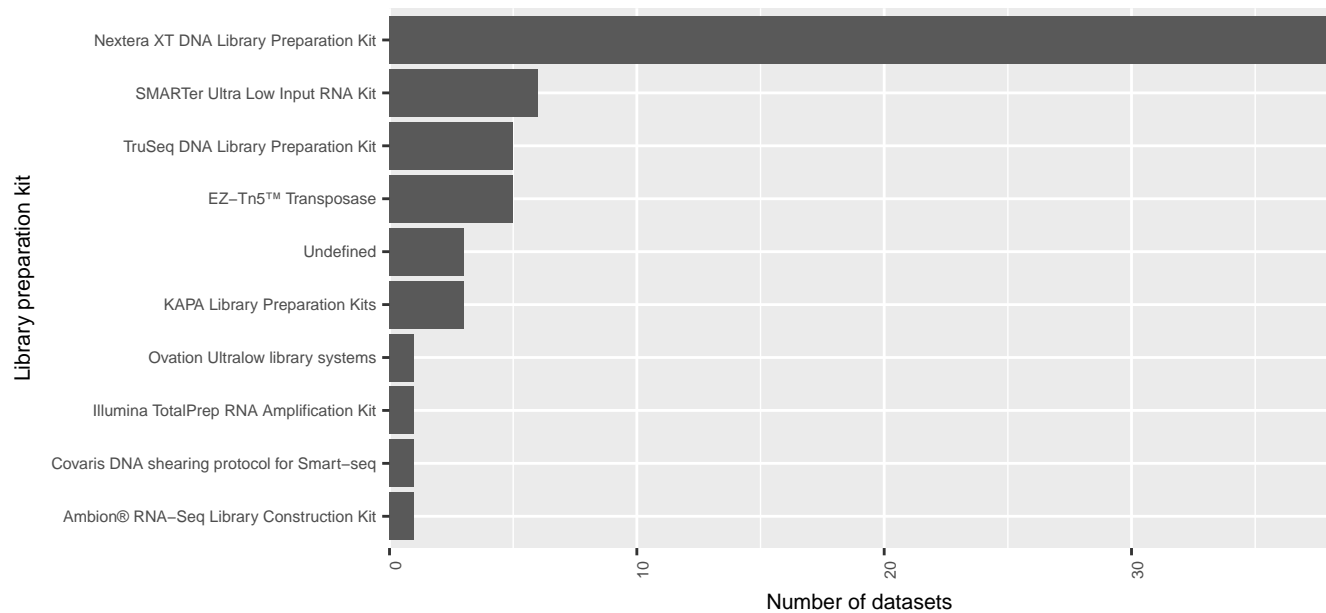

Supplement: Supplementary Data [file gkx949_supp.zip › nar-02451-data-e-2017-File014.pdf]

Total number of single-cells Homo sapiens

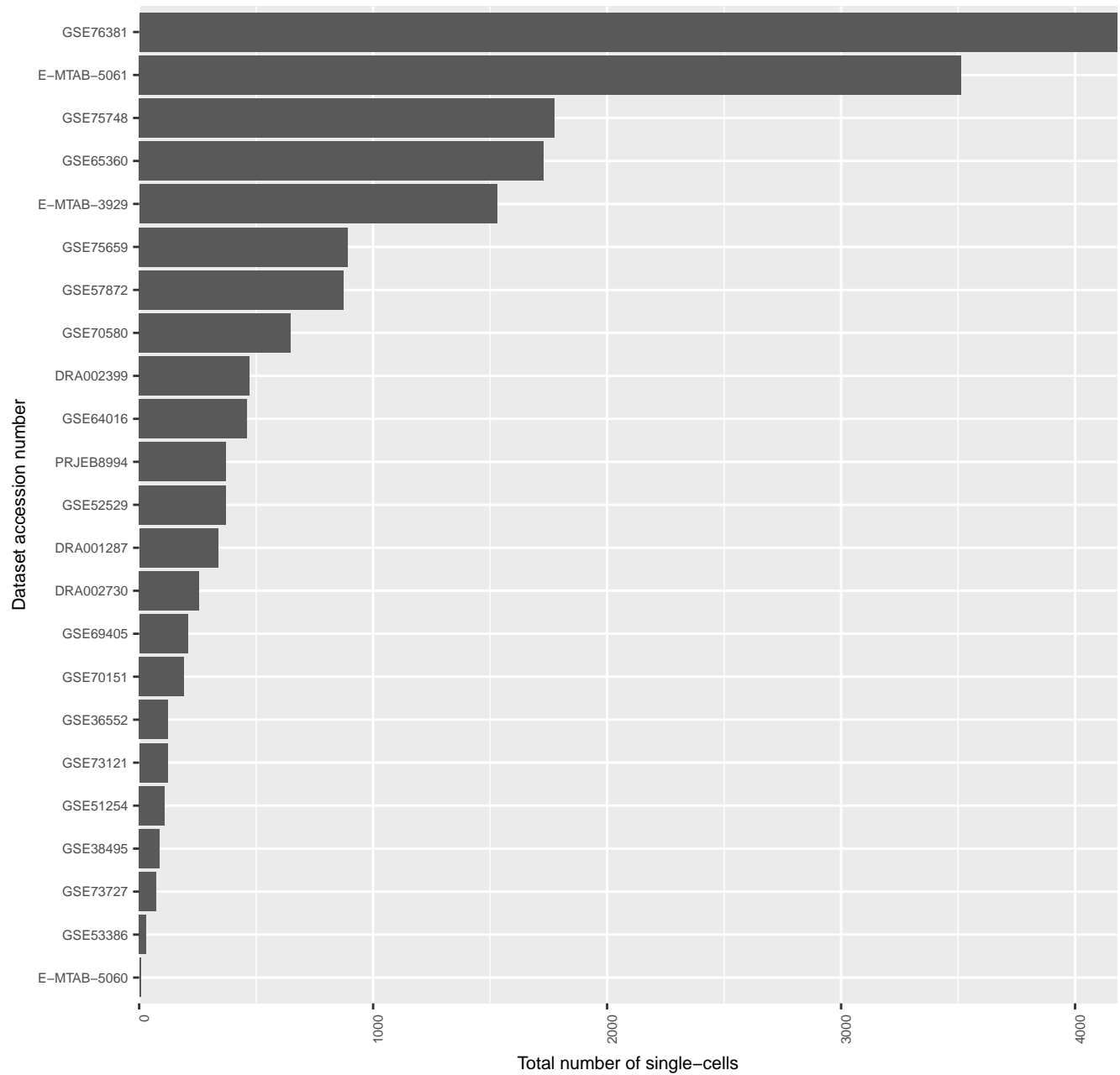

Supplement: Supplementary Data [file gkx949_supp.zip › nar-02451-data-e-2017-File015.pdf]
